# How does a motor or cognitive dual-task affect our sense of upper limb proprioception?

**DOI:** 10.1371/journal.pone.0299856

**Published:** 2024-03-20

**Authors:** Amanda L. Ager, Ann M. Cools, Dorien Borms, Jean-Sébastien Roy

**Affiliations:** 1 Center for Interdisciplinary Research in Rehabilitation and Social Integration (CIRRIS), Quebec City, Quebec, Canada; 2 Department of Rehabilitation Sciences and Physiotherapy, Faculty of Medicine and Health Sciences, Ghent University, Ghent, Belgium; 3 Department of Human Structure and Repair, Faculty of Medicine and Health Sciences, Ghent University, Ghent, Belgium; 4 School of Rehabilitation Sciences, Faculty of Medicine, Université Laval, Quebec City, Quebec, Canada; UFPE: Universidade Federal de Pernambuco, BRAZIL

## Abstract

**Background:**

Daily upper limb activities require multitasking and our division of attention. How we allocate our attention can be studied using dual-task interference (DTi). Given the vital role proprioception plays in movement planning and motor control, it is important to investigate how conscious upper limb proprioception is impacted by DTi through cognitive and motor interference.

**Purpose:**

To examine how dual-task interference impacts conscious upper limb proprioception during active joint repositioning tasks (AJRT).

**Methods:**

Forty-two healthy participants, aged between 18 and 35, took part in this cross-sectional study. Participants completed two AJRT during three conditions: baseline (single task), dual-cognitive task (serial subtractions), and dual-motor task (non-dominant hand movements). The proprioceptive error (PE; difference between their estimation and targeted position) was measured using an AJRT of 75% and 90% of maximum internal rotation using the Biodex System III^TM^ and the Upper Limb Proprioception Reaching Test (PRO-Reach). To determine if PEs differed during dual-task interference, interference change scores from baseline were used with one sample *t*-tests and analyses of variance.

**Results:**

The overall mean PE with the Biodex was 4.1° ± 1.9 at baseline. Mean change scores from baseline reflect a mean improvement of 1.5° ± 1.0 (*p* < .001) during dual-cognitive task and of 1.5° ± 1.2 (*p* < .001) during dual-motor task. The overall mean PE with the PRO-Reach was 4.4cm ± 1.1 at baseline. Mean change scores from baseline reflect a mean worsening of 1.0cm ± 1.1 (*p* < .001) during dual-cognitive task and improvement of 0.8cm ± 0.6 (*p* < .001) during dual-motor task. Analysis of variance with the Biodex PEs revealed an interference effect (*p* < .001), with the cognitive condition causing greater PEs compared to the motor condition and a criterion position effect (*p* = .006), where 75% of maximum IR produced larger PEs during both interference conditions. An interference effect (*p* = .022) with the PRO-Reach PEs was found highlighting a difference between the cognitive and motor conditions, with decreased PEs during the contralateral motor task.

**Conclusion:**

Interference tasks did impact proprioception. Cognitive interference produced mixed results, whereas improved proprioception was seen during motor interference. Individual task prioritization strategies are possible, where each person may choose their own attention strategy when faced with dual-task interference.

## Introduction

Proprioception is a sensorimotor sense that our body relies on for safe and purposeful movements while navigating our surroundings [1]. It is involved in guiding precise movement patterns, balance and joint stability, and thus provides vital feedback about changes in the conditions of movement [2, 3]. This is relevant to the upper limbs, particularly to the glenohumeral (GH) joint, due to the sacrifice of joint stability [4] for a large range of motion [5, 6]. Proprioception contributes to dynamic shoulder joint stability during upper limb activities such as sports [7, 8], playing a musical instrument [9] and in daily activities (ADLs) [10].

There is both conscious (cerebral) and unconscious (peripheral and cerebellar) levels of processing of proprioceptive information. Conscious proprioception contributes to the activation of muscles during voluntary movements, whereas unconscious proprioception enables us to subconsciously monitor the positioning of our body [11]. Conscious proprioception can be understood as our awareness within our environment, which accompanies the perception of the environment; hence the concepts of perception, consciousness and proprioception are intimately linked [12, 13]. For the purpose of this paper, any mention of proprioception is referring to the conscious processing of proprioception information, as the framework behind unconscious proprioception is not fully understood [14, 15].

It is important to acknowledge that proprioceptive information feeds into the multicomponent sensory system [11], along with competing senses and cognitive processes such as attention, in order to make decisions regarding our movements [16]. Indeed, proprioception is considered to be interoceptive, meaning that its sensory inputs are derived from changes within internal structures [17]. This is worth considering when appreciating how proprioceptive information is combined with other sensory inputs (visual, vestibular, tactile, spatial orientation, for example) while also being integrated alongside higher cerebral mechanisms such as memory and attention.

Sensory proprioceptive information is prioritized alongside other sensorimotor and cognitive processes, such as our allocation of attention [18], in order to effectively perform our ADLs [19]. While much of our daily bodily movements are automated, acquiring complex motor skills does necessitate conscious attention [20]. Examples of upper limb movements, such as throwing or catching a ball, performing arm movements during ice skating, and engaging in coordinated patterns in Tai Chi, all demand an aspect of conscious awareness [20]. Despite the evident conscious aspect to motor control, the impact of how we allocate our attention while also integrating sensory information, including proprioception, to generate purposeful movements remains poorly understood. How we prioritize our sensory information and attention during two or more concurrent tasks is therefore important for clinicians and researchers alike to understand, as it may lead to new motor learning or rehabilitation approaches for sensory integration and movement planning during dual-task activities. In this view, dual-task interference (DTi) is a popular scientific method for understanding our allocation of attention when performing one task while being engaged in another [21]. During multi-tasking, there can be impairments in the performance of one, or both, of the simultaneous tasks (mutual interference) [22, 23].

DTi has been studied in the lower limbs in terms of movement kinematics [22] and in relation to gait and postural control [19, 24]. The influence of DTi on sensory processes has also been studied with postural sway and stability [24], visual attention [25] and conscious proprioception [19, 22]. However, there is limited evidence regarding these effects involving the upper limbs [26, 27]. Upper limb multitasking has become central to today’s complex work environment [28], whether that includes manual labour or working from a computer. Given the important role proprioception plays throughout upper limb movement planning and motor control [3], it is relevant to investigate how our sense of upper limb proprioception may be impacted by DTi [29, 30].

A recent study by Jiang et al. (2023) investigated how DTi, through a cognitive task (serial subtractions), affected active and passive knee joint position sense during a leg drop test [22]. The authors suggested that dual-tasking reduces motor performance through the slowing down of proprioceptive information processing, without affecting movement execution. It has been proposed that the greater the attentional demands of a task, the poorer the proprioception accuracy of our movements [31, 32]. Yet, the effects of dual-task performance on proprioceptive information processing is not well understood [22]. Therefore, the purpose of this study was to investigate in healthy participants how DTi (cognitive interference [dual cognitive task] and motor interference [dual contra-lateral motor task]) impacts ipsilateral conscious upper limb proprioception, more precisely active joint position sense (AJPS), a sub-category of proprioception. The evaluation of AJPS most closely resembles upper limb functional movements while maximising the ecological validity [33] of proprioception testing [34]. Furthermore, this study will consider whether the magnitude of proprioception or attentional errors will be most affected by DTi, or if they will both be equally affected through a mutual interference effect [22, 23].

We hypothesized that both cognitive and motor interference would induce a significantly greater proprioception error (PE) compared to baseline (single task). It is the current view that movement outcomes are on a continuum from most to least automatic, and that automatic-like motor responses have low attentional costs [35]. Accordingly, we further hypothesized that the cognitive interference would require more mental effort [23] and will induce a greater PE than the motor interference, since the motor dual-task is anticipated to have a lower attentional cost [36]. Lastly, we anticipate a strong and positive correlation between the magnitude of proprioception errors and the number of attentional errors made during DTi, due to an effect of mutual interference.

## Materials and methods

### Study design

A cross-sectional, single-session design (150-minutes) was used. Participants performed two active joint repositioning tasks (Biodex System III [Biodex] and the Upper Limb Proprioception Reaching Test [PRO-Reach]) under three conditions: baseline (no interference), cognitive interference and motor interference. Prior to proprioception testing, the attentional errors during both cognitive and motor interference tasks were also assessed to establish a baseline measurement for each interference task per participant.

### Participants

Healthy participants from Ghent University, Belgium were recruited (between 01/07/2019 and 15/09/2019) if they respected the following: 1) were between the ages of 18 and 35; 2) reported no musculoskeletal injuries to either the upper limbs or cervical-thoracic spine within the past two years; and 3) had no other major health concerns (e.g., signs, symptoms or diagnosis of a systemic or neurological pathology) that would prevent them from participating in the study. The *Quick* Disability of the Arm, Shoulder, or Hand (*Quick*DASH score; 0 –no disability, 100 –most severe disability) questionnaire [37] was used to ensure participants did not report any upper limb functional limitations. The *Quick*DASH has a minimal clinically important difference (MCID) of 15.9 points, which was used as the inclusion threshold. Participants were recruited through face-to-face communication or e-mail. All gave informed and written voluntarily consent for participation. This study was approved by the Ethical Committee of Ghent University, Registration Number: B8670201940272.

The G*Power (version: 3.1.9.7) software was used to determine the sample size. Based on previous interference studies [38, 39] effect size was set to large (η_p_^2^  = .14). In order to achieve an alpha of .05 and a power of .95, the required minimum sample size is 35 for a repeated measures ANOVAs with seven levels.

### Proprioception outcome measures

AJPS of the dominant upper limb was measured using an isokinetic dynamometer (Biodex Multi-Joint System III; Biodex Medical Systems, Inc., Shirley, NY, USA) [Biodex] [40] and the Upper Limb Proprioception Reaching Test (PRO-Reach) [41]. Although there is no accepted “Gold Standard” for measuring shoulder proprioception [20, 42, 43], a recent systematic review [34] suggests that isokinetic dynamometer supports the highest reliability for measuring shoulder JPS (weighted average intra-session ICC = 0.92 ± 0.07) [34]. However, the tool is not always accessible to clinicians and the proprioception shoulder protocol with the Biodex only evaluates one degree of freedom, which does not accurately represent every day functional movements. For these reasons, as well as to increase the ecological validity of proprioception testing, the PRO-Reach tool was added. Recent psychometric testing of the PRO-Reach supports an overall good level of intra-rater reliability (ICC = 0.77) [41].

### AJPS with Biodex

The Biodex was used with the upper limb Biodex arm attachment. Participants were seated and secured with pelvic and torso straps. The GH joint was positioned at 90° abduction, 0° of rotation with 90° of elbow flexion. Internal rotation (IR) was evaluated, as it is often used to quantify shoulder JPS, [5, 44, 45] and is a reliable movement with the Biodex (ICC = 0.88) [34]. After determining the maximal (max) active range of motion (AROM) for IR, 75% and 90% of their max AROM was calculated for relative target angles (criterion positions). Relative target angles were used to take into account each participant’s movement ranges, while representing the same stretching of the soft tissues, thus activating mechanoreceptors to the same degree for each participant [45]. Before testing, the system was calibrated according to the manufacturer’s guidelines [40].

Participants were given three mid-range familiarization trials at 50% of max IR; consisting of three memorization trials, where participants were asked to actively move their shoulder to the target angle with the Biodex arm and “*memorize the position of their arm in space*” for 5 seconds [46]. Followed by three reproduction trials, where participants were asked to move actively towards the target and reproduced the memorized position. Instructions included “*Ready*, *Go*, *Hold*, *Relax*”. Participants were asked to indicate their estimated position, by clicking the "stop button”, where the PE was recorded in degrees. A mid-range practice target angle, 50% of their max IR, was chosen to minimize any possible learning effect with the end-range target angles used during the evaluation. Participants were blindfolded for all trials. Following the familiarization trials, each participant was evaluated at 75% and 90% of max IR, producing three PEs per criterion position.

### AJPS with the PRO-Reach

The PRO-Reach is plasticized poster (90 cm x 110 cm), mounted on a wall with double-sided magnetic strips (ProMAG® Magnetic Tape; Marietta, OH, U.S.A.). The PRO-Reach uses stickers (0.6 cm round, manually numbered 1–3) to mark the three unconstrained reaching trials per target. The purpose is to “*evaluate the ability to reproduce movements in space”* and participants are instructed to *“memorize the position of your arm in space*".

Seven targets were used and are named according to the direction of movement of the dominant shoulder. For example, the left-side of the PRO-Reach is dominant for left-handed participants, and the right-side of the PRO-Reach represent non-dominant (ND) cross-body movements, and vice-versa for right-handed participants. The targets are named: superior (S), superior-lateral dominant (SLD) and non-dominant (SLND), lateral-dominant (LD) and non-dominant (LND) and inferior-lateral dominant (ILD) and non-dominant (ILND). The (S) target is used to evaluate both right and left-handed participants. Three familiarization trials (three memorization and reproduction trials) were practiced using the superior target.

**Fig 1:**
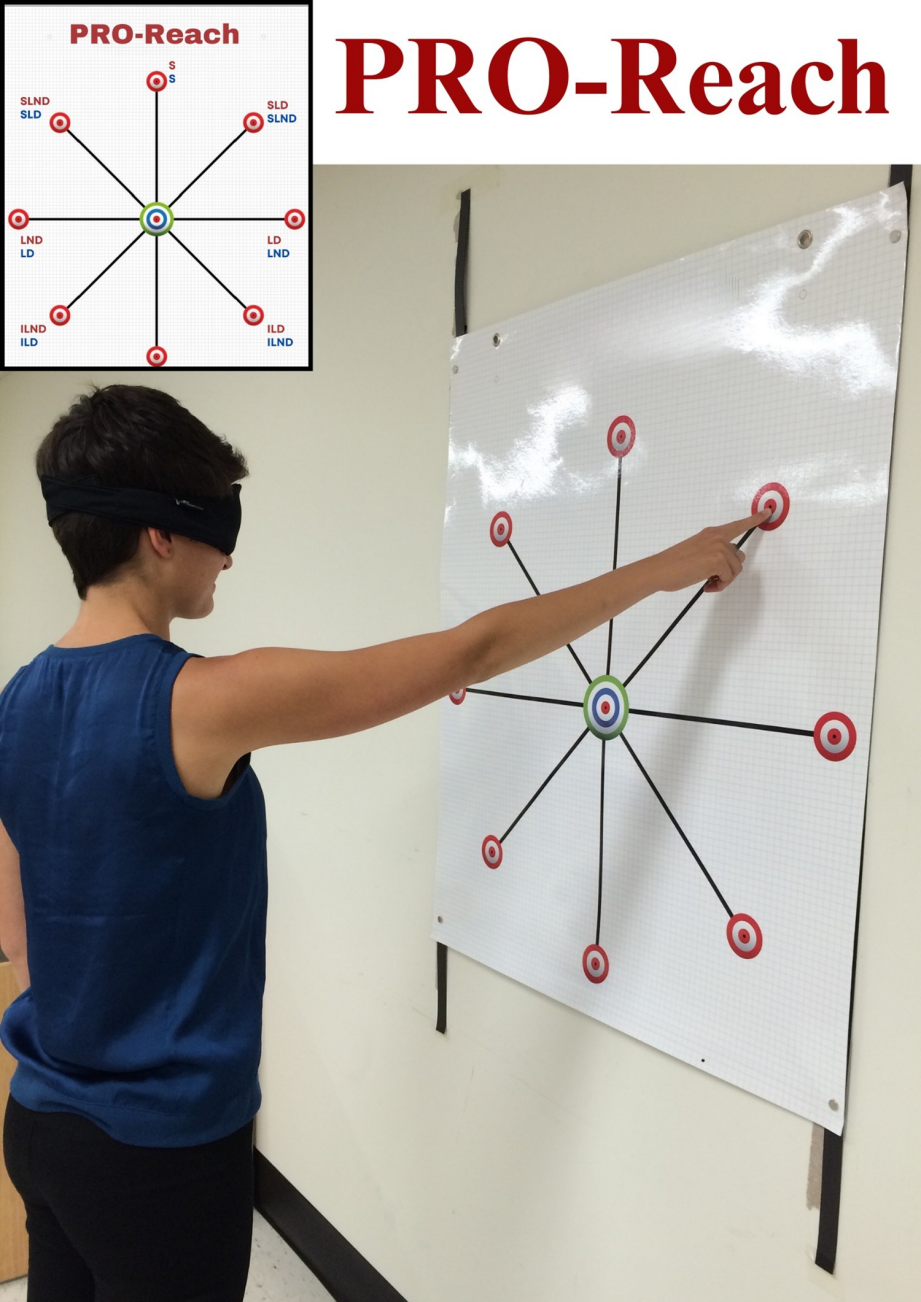
Laboratory set up of the shoulder Proprioception Reaching Test (PRO-Reach) for the evaluation of active joint position sense of the dominant upper limb, with reaching movements towards targets in standing. Proprioception error in centimetres (cm). The individual pictured in Fig 1 has provided written informed consent, as outlined in PLOS consent form, to publish their image alongside the manuscript.

The participants performed three memorization trials, where they reached with their eyes open towards an indicated target. Once their index finger reached the centre of the target, they would close their eyes and memorize their position in space for 5 seconds. Following the memorization trials, they would promptly apply a blind fold with their non-dominant hand, and immediately perform three reproduction reaching trials towards the target (used to measure the PEs) (Fig 1). No feedback or corrections are given during testing. Three PEs were recorded per target. See *Supporting Information (S1 File)* for the complete PRO-Reach evaluation protocol.

### Dual-task interference

#### Cognitive interference

Cognitive interference consisted of serial subtractions from 100, by multiples of three. A “cognitive error” consisted of i) an error in numbering, ii) a word whisker (such as Umm, Errr etc.), or iii) a pause longer than 2 seconds during counting, which was confirmed by the consensus of the two evaluators quantifying the cognitive errors.

#### Motor interference

Motor interference consisted of a contralateral (non-dominant) hand movement. Participants were asked to open their hand and digits as far as possible and close them again to make a fist. No specific guidance was provided for speed of movement. “Motor errors” consisted of i) the participant not fully extending or flexing their digits ii) the participant stopped moving their hand or digits for 2 seconds or longer, which was confirmed by the consensus of the two evaluators quantifying the motor errors.

The mean number of attentional errors (frequency counts) during cognitive and motor interference were recorded by two evaluators (L. Li, & L. Le). At the end of laboratory session, participants were asked “*Which task did you find more difficult*, *counting backwards by threes*, *or opening and closing your hand while performing the other task*?”.

#### Randomization and rest periods

The order of the outcome measures (Biodex or PRO-Reach), criterion positions for the Biodex and targets for the PRO-Reach, as well as the order of the dual-tasks (cognitive or motor) were randomized for each participant, using an online list randomizer (https://www.random.org/lists/) by an independent evaluator. Each participant was given 10 minutes of rest between each testing condition and between outcomes.

#### Evaluators

Four evaluators were needed. The first evaluator (A.L.A) explained the study and obtained consent, evaluated handedness, collected anthropometric measurements, directed the evaluation sessions and recorded the PEs. Handedness was determined by asking “*Which arm do you use to throw a ball*?”. If the answer was both or ambiguous, the Edinburgh Handedness Inventory questionnaire—Short Form [47] was issued. The second evaluator measured the PEs with the Biodex and PRO-Reach, whereas the third and fourth evaluators counted the attentional errors.

#### Data analysis

The proprioception variables included the **absolute PEs** from the active joint repositioning tasks with the Biodex (degrees for each criterion position) and PRO-Reach (displacement in centimetres per target). The absolute PE represents the difference between the participant’s estimation and the targeted position. Each criterion position (Biodex) and target (PRO-Reach) produced three PEs, the **absolute mean** per criterion position and target were used for descriptive analysis. Also, the **global mean PEs** (the average of the means per targets [PRO-Reach]) were used to represent an overall proprioception score for the PRO-Reach. The Biodex evaluation produced an absolute mean per criterion position (75% and 90% of max IR), per condition (baseline / cognitive / motor) for a total of six data points for each participant. The PRO-Reach evaluation produced an absolute mean per target (7 targets), per condition (baseline / cognitive / motor), for a total of 21 data points for each participant.

For the interference tasks, the cognitive and motor variables included the number of cognitive and motor errors made during baseline and dual-task interference. The mean errors counted between the two evaluators were used for statistical analysis.

#### Statistical analysis

Descriptive statistics (means ± standard deviations) were calculated across participants for all variables i.e., the absolute PEs and the number of attentional errors made during all three conditions (baseline / cognitive / motor). All data was tested to verify the distributional assumptions for the inferential statistical analyses.

To evaluate the change in PE between baseline and cognitive interference (Baseline PE–Cognitive PE), and baseline and motor interference (Baseline PE–Motor PE), participants acted as their own controls and change scores were used, producing an **interference change score from baseline**. To indicate the directionality of the PE change score, a negative PE indicates a worsening of proprioception, and a positive PE indicates an improvement of proprioception, compared to baseline (single task). To examine whether participants differed from their baseline performance during cognitive and motor interference, one-sample *t*-tests with bootstrapping were performed using the change scores. For the purpose of this study, (μ = 0) indicates that the mean change scores were compared with a theoretical mean of zero.

To determine if the effects differed according to the conditions of measurement, repeated-measures analysis of variances (ANOVAs) were performed. The distribution of the data was visually confirmed by examining frequency distributions through histograms, as well as by corrected Quasi Likelihood under Independence Model Criterion (QICC) and goodness of fit tests. Mauchly’s test of sphericity was used to assess the assumption of sphericity for repeated measures ANOVAs. Partial Eta Squared (η^2^) were calculated to estimate the effect sizes between variables, where η^2^ = 0.01 indicates a small effect, η^2^ = 0.06 indicates a medium effect and η^2^ = 0.14 indicates a large effect. Using the interference change scores from baseline, the analyses consisted of a 2×2 (interference [cognitive / motor] X criterion positions [75% and 90% of max IR]) for the Biodex and a 2×7 (interference [cognitive / motor] X targets) analyses for the PRO-Reach. To analyse whether the proprioception errors differed between interference conditions (cognitive and motor), interference change scores were analysed through *post hoc* paired *t*-tests for both outcomes. **Estimated marginal means (EMMs)** were calculated with Bonferroni corrections to verify any main effects against the corresponding simple effects. A marginal mean is the mean response for each category of a factor, adjusted for any other variables in the linear model. In some circumstances, descriptive means and marginal means are equal, however in some cases they can differ. This is due to descriptive means being based solely on the observed data, whereas marginal means are estimated based on a statistical model. The estimated marginal means for the proprioception errors were obtained by averaging the marginal means across interference conditions.

To analyse the relationship between the attentional errors (cognitive and motor) and the PEs, Pearson correlation coefficients were employed, as all variables were normally distributed. The Pearson correlation coefficient (*r*) was categorized as weak (≤0.499), moderate (0.50–0.707), or strong (≥0.707) [48].

Intraclass correlation coefficients (ICCs) were used to assess the inter-rater reliability between the two evaluators for their frequency counts of the attentional errors. ICC(_3,k_) (two-way random model with absolute agreement) were calculated. ICC values less than 0.5 indicate poor reliability, values between 0.5 and 0.75 indicate moderate reliability, values between 0.75 and 0.90 indicate good reliability, and values greater than 0.90 indicate excellent reliability [49]. All statistical analysis was conducted using IBM SPSS® Software (version 26.0 for Mac; Armonk, NY) with an alpha level of 0.05.

## Results

Forty-five healthy individuals were recruited, three were excluded due to upper limb symptoms, resulting in 42 participants. Of the 42 healthy participants, 13 were male and 29 females, with a mean age of 22.9 years (± 2.0 years), mean weight of 70.9 kg (± 13.7 kg), with no reported functional limitations (all *Quick*DASH scores were equal to zero). Thirty-eight participants reported being right-handed (90%) and four left-handed (10%). Thirty-six (85.7%) participants found the cognitive interference task to be more difficult, whereas six (14.3%) indicated the motor interference task to be more difficult. A high level of agreement between the two evaluators was found for counting of the attentional errors (cognitive ICC range = 0.86–0.90, motor ICC range = 0.85–0.95).

### Proprioception errors (PEs) during DTi

The one-sample *t*-tests used to examine whether participants differed from their baseline performance during cognitive and motor interferences (interference change score from baseline) can be found in Table 1. The overall mean PE with the Biodex (in degrees) was 4.1 ± 1.9 at baseline. Mean change scores from baseline reflect a mean improvement of 1.5 ± 1.0 (*p* = < .001) during dual-cognitive task and of 1.5 ± 1.2 (*p* = < .001) during dual-motor task. The overall mean PE with the PRO-Reach (in centimeters) was 4.4 ± 1.1 at baseline. Mean change scores from baseline reflect a mean worsening of 1.0 ± 1.1 (*p* = < .001) during dual-cognitive task and improvement of 0.8 ± 0.6 (*p* = < .001) during dual-motor task.

**Table 1:** Analysis of the change of the proprioception errors compared to baseline using one-sample *t*-tests.

	Cognitive		Motor	
	Mean PE change from baseline mean (95%CI)	*t*-tests	Mean PE change from baseline mean (95%CI)	*t*-tests
**Biodex System (change from baseline in°)**				
**AJPS 75%**	+3.6 [2.9 to 4.4]	***p* = < .001** *	+2.4 [1.8 to 3.1]	***p* = < .001** *
**AJPS 90%**	+1.3 [0.9 to 1.7]	***p* = .001** *	+1.1 [0.9 to 1.3]	***p* = < .001** *
**PRO-Reach (change from baseline in cm)**				
***S***	−1.9 [1.5 to 2.5]	***p* = < .001** *	+1.6 [1.3 to 1.9]	***p* = < .001** *
***SLD***	+1.8 [1.3 to 2.3]	***p* = < .001** *	+1.6 [1.2 to 2.0]	***p* = < .001** *
***LD***	−2.1 [1.6 to 2.7]	***p* = < .001** *	−1.9 [1.4 to 2.4]	***p* = < .001** *
***ILD***	−2.2 [1.3 to 3.0]	***p* = < .001** *	−2.1 [1.5 to 2.7]	***p* = < .001** *
***SLND***	+1.91 [1.5 to 2.4]	***p* = < .001** *	+1.8 [1.5 to 2.2]	***p* = < .001** *
***LND***	−2.5 [1.9 to 3.1]	***p* = < .001** *	+1.9 [1.4 to 2.5]	***p* = < .001** *
***ILND***	−2.5 ± 2.4 [1.8, 3.2]	***p* = < .001** *	+1.9 [1.4 to 2.4]	***p* = < .001** *
**Global mean** (Seven targets)	−1.0 [0.7 to 1.3]	***p* = < .001** *	+0.8 [0.6 to 1.0]	***p* = < .001** *

This table represents the change in PE between baseline and cognitive interference (baseline PE–cognitive PE), and baseline and motor interference (baseline PE–motor PE); where participants acted as their own controls and change scores were used.

A negative value indicates a worse PE, while a positive value indicates an improved PE, compared to baseline (no interference). One sample *t*-tests were conducted between baseline (no interference) vs. cognitive interference, and baseline (no interference) vs. motor interference.

AJPS = active joint position sense, PRO-Reach = Upper Limb Proprioception Reaching Test

Biodex System = Isokinetic Dynamometer, Biodex System III.

95%CI = 95% confidence interval [lower limit to upper limit].

Descriptive statistics (Proprioception Errors [PE] in degrees [Biodex System] and in centimeters [PRO-Reach]) of healthy participants (n = 42). Mean ± 95%CI [lower limit–upper limit] of the PE.

(*) Statistically significant results. PRO-Reach targets: Superior (S), Superior Lateral Dominant (SLD), Lateral Dominant (LD), Inferior Lateral Dominant (ILD), Superior Lateral Non-Dominant (SLND), Lateral Non-Dominant (LND) and Inferior Lateral Non-Dominant (ILND).

### Analysis of variance for the proprioception and attentional errors during DTi

#### Biodex (PEs in degrees)

**Fig 2:**
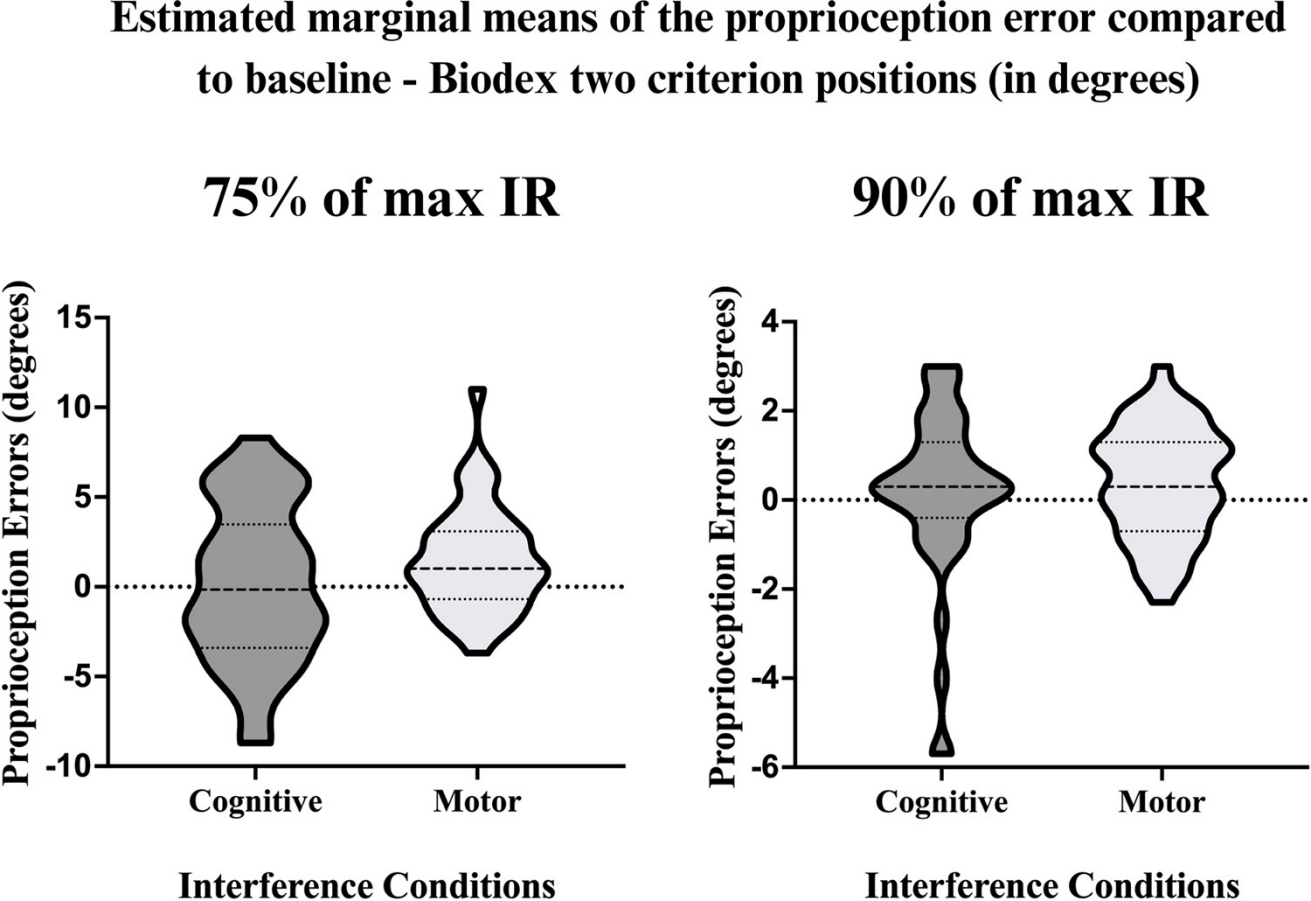
Estimated marginal means of the proprioception error compared to baseline—Biodex two criterion positions (in degrees). This estimated marginal means graph represents the change in PE between baseline and cognitive interference (baseline PE–cognitive PE), and baseline and motor interference (baseline PE–motor PE); where participants acted as their own controls. A marginal mean is the mean response for each category of a factor, adjusted for any other variables in the linear model. The estimated marginal means for the proprioception errors were obtained by averaging the marginal means across interference conditions. A negative value indicates a worse PE, while a positive value indicates an improved PE, compared to baseline (no interference). Evaluated with the Biodex outcome, proprioception acuity improved under both the cognitive and motor conditions.

The ANOVAs performed using the interference change scores from baseline (two criterion positions) and two interference conditions (cognitive and motor), did not reveal a significant **interaction effect** (criterion position × interference) (*F* (1.41) = 3.2, [*p* = .083], η^2^ = .071). However, a significant **main criterion position effect** (*F* (1.41) = 8.4, [*p* = .006], η^2^ = .169) was found, with the 75% of max IR criterion position producing a larger PE under both the cognitive ($\bar{X}$ = 3.6 ± 0.4 [2.8 − 4.4]) and motor ($\bar{X}$ = 1.3 ± 0.2 [0.9 − 1.7]) conditions, compared to the 90% of max IR criterion position for the cognitive ($\bar{X}$ = 2.4 ± 0.3 [1.8 − 3.1]) and motor ($\bar{X}$ = 1.1 ± 0.1 [0.9 − 1.3]) conditions. Moreover, an **interference effect** was found (*F* (1.41) = 33.7, [*p* < .001], η^2^ = .451), indicating a significant difference between the cognitive and motor interference conditions. *Post-hoc* analyses indicate that proprioceptive acuity was significantly better at 75% of max IR during the cognitive interference condition compared to motor conditions (mean difference of 1.2 [± 0.2] degrees; *p* = .006). There was not a significant difference between the two interference conditions at 90% of max IR (mean difference of 0.2 [± 0.7] degrees; *p* = .18). Fig 2 demonstrates the difference between the PEs during the cognitive and motor conditions through EMMs for both criterion positions with the Biodex.

#### PRO-Reach (PEs in centimeters)

**Fig 3:**
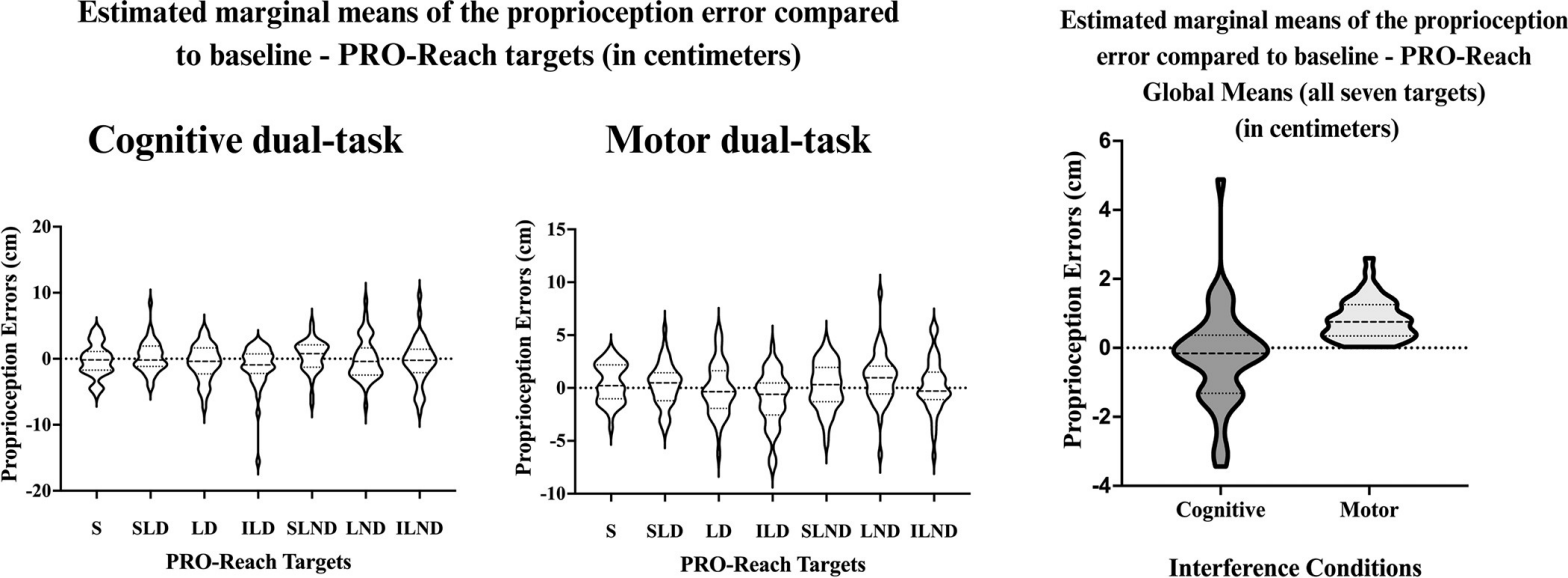
Estimated marginal means of the proprioception error compared to baseline—PRO-Reach global means (in centimeters) this estimated marginal means graph represents the change in PE between baseline and cognitive interference (baseline PE–cognitive PE), and baseline and motor interference (baseline PE–motor PE); where participants acted as their own controls. A marginal mean is the mean response for each category of a factor, adjusted for any other variables in the linear model. The estimated marginal means for the proprioception errors were obtained by averaging the marginal means across interference conditions. A negative value indicates a worse PE, while a positive value indicates an improved PE, compared to baseline (no interference). Evaluated with the PRO-Reach outcome, proprioception acuity worsened under cognitive interference but improved under the motor interference.

The ANOVAs performed using the interference change scores from baseline with the PRO-Reach (seven targets) and two interference conditions (cognitive and motor), did not reveal a significant **interaction effect** (targets × interference) (*F* (6.25) = .52, [*p* = .80], η^2^ = .012). Moreover, no **main target effect** (seven targets) was observed (*F* (6.25) = .94, [*p* = .47], η^2^ = .022), but a significant **main interference effect** (*F* (1.41) = 5.7, [*p* = .022], η^2^ = .122) was found, indicating a significant difference between the cognitive and motor interference conditions. *Post hoc* comparisons between the cognitive and motor conditions for each PRO-Reach target found that proprioception acuity was better with the LND (mean difference of 0.6 [± 1.8] cm; *p* = .006) and ILND (mean difference of 0.6 [± 2.0] cm; *p* = .04) targets under the motor condition compared to the cognitive condition. However, the remaining targets (S / SLD / LD / ILD/ SLND) do not support a significant difference between the cognitive and motor conditions (*p* > .05). The global mean PEs of the PRO-Reach (all seven targets) suggests a mean difference of 3.9 ± 0.1 cm (*p* = .04) between conditions with better proprioception acuity during the motor condition. Fig 3 demonstrates the difference between the global mean PEs (all seven targets) during the cognitive and motor conditions through EMMs for the PRO-Reach.

#### Attentional errors during DTi

The mean count of attentional errors made during dual-task testing can be found in Table 2. Attentional errors made during the evaluation with the Biodex for the cognitive (range from 0.0 to 4.0 errors) and motor (range from 0.0 to 4.7 errors) conditions for both criterion positions remained low. The same can also be said about the mean number of attentional errors made during the evaluation with the PRO-Reach, during the cognitive (range from 0.0 to 1.0 errors) and motor (range from 0.0 to 3.2 errors) interference conditions.

**Table 2:** Mean count of attentional errors made during dual-task testing.

Proprioception Outcome	Cognitive Errors	Motor Errors
	mean ± SD	mean ± SD
	median (IRQ)	median (IRQ)
**Biodex System (AJPS errors in°)**		
**AJPS 75%**	0.9 ± 0.9	1.3 ± 1.3
	0.8 (0.9)	0.9 (1.4)
**AJPS 90%**	0.7 ± 0.7	1.3 ± 1.5
	0.5 (0.8)	0.8 (1.6)
**PRO-Reach (AJPS errors in cm)**		
**S**	0.3 ± 0.4	0.8 ± 0.8
	0.3 (0.5)	0.8(1.0)
**SLD**	0.3 ± 0.3	0.8 ± 0.6
	0.2 (0.5)	0.7 (0.7)
**LD**	0.3 ± 0.4	0.7 ± 0.8
	0.0 (0.3)	0.4 (1.0)
**ILD**	0.4 ± 0.4	0.7 ± 0.6
	0.3 (0.6)	0.7 (0.7)
**SLND**	0.3 ± 0.4	0.8 ± 0.8
	0.1 (0.6)	0.7 (0.9)
**LND**	0.4 ± 4.5	0.9 ± 0.9
	0.3 (0.6)	0.8 (0.9)
**ILND**	0.3 ± 0.4	0.8 ± 0.9
	0.3 (0.7)	0.7 (1.2)
**Global mean**	0.3 ± 0.1	0.8 ± 0.7
**(7 targets)**	0.3 (0.4)	0.7 (0.7)

Baseline attentional errors assessed before the evaluation of proprioception (no proprioception interference) and during proprioception testing (dual-task interference).

The cognitive task comprised of counting backwards from 100 by threes. An error was counted if the participant did not state the correct number, if there was a word whisker (errr, ummm), or if there was a pause longer than 2 seconds.

The motor task included opening and closing of their non-dominant hand. An error was counted if the participant was unable to fully extend or flex their digits or thumb, or if their movements stopped for longer than 2 seconds. The mean number of attentional errors, as assessed by two evaluators, during the simultaneous proprioception testing of the Biodex or PRO-Reach (dual-task interference).

AJPS = active joint position sense, PRO-Reach = Upper Limb Proprioception Reaching Test

Biodex System = Isokinetic Dynamometer, Biodex System III.

Descriptive statistics of attentional errors, $\bar{X}$ = mean ± SD = standard deviation, median & interquartile range (IQR) of healthy participants (n = 42). PRO-Reach targets: Superior (S), Superior Lateral Dominant (SLD), Lateral Dominant (LD), Inferior Lateral Dominant (ILD), Superior Lateral Non-Dominant (SLND), Lateral Non-Dominant (LND) and Inferior Lateral Non-Dominant (ILND).

No correlational relationship was found between the PEs and the attentional errors produced during the cognitive or motor interference conditions, for any of the proprioception outcomes (*r* ranges from, .095 − .284, *p* > .05) (*Supporting Information*). Likewise, no significant correlations were found between baseline errors (single task) and cognitive and motor attentional errors made during proprioceptive testing (dual-task) (*p* > .05). Descriptive statistics of the attentional errors can be found in the *Supporting Information*.

## Discussion

The purpose of this study was to investigate how dual-task interference would impact the AJPS of healthy upper limbs, in an effort to understand the sensorimotor factors which could affect conscious proprioception. Our results suggest that upper limb AJPS is influenced by dual-task interference, although not as originally hypothesized. With significant interference effects with both the Biodex and PRO-Reach outcomes, conscious upper limb proprioception is influenced by dual-task conditions, regardless of the measurement outcome or targeted position. The cognitive interference condition had a mixed impact on conscious proprioception, with the Biodex evaluating lower PEs (improved proprioception) and the overall global mean of the PRO-Reach outcome demonstrating higher PEs (worse proprioception) compared to baseline. The motor interference condition demonstrated a stronger tendency for lower PEs (improved proprioception) for the global proprioception outcomes for both the Biodex and PRO-Reach.

### How is proprioception affected by dual-interference?

Our study found mixed results as to the effects of a dual cognitive interference condition on upper limb AJPS. A study by Jiang and colleagues [22] involving the lower limbs examined the effects of a cognitive dual-task interference on knee JPS. Their results found that dual-tasking reduces the speed at which proprioception information is processed, but does not have a significant impact on knee JPS itself [22]. It is interesting to note that their study did not evaluate the effects of a dual-motor task on proprioception. Previous studies have employed a cognitive-motor dual-task paradigm, which investigated how a cognitive task impacted a motor performance outcome such as balance, body sway, gait kinematics, step length, reaction time or joint velocity [50–52]. Such studies have predominantly focused on the lower limbs [22]. At present, the comparison of our results to the current literature is not possible, as the influence of a dual motor task on conscious proprioception with the upper limbs has not been adequately investigated. Nevertheless, our findings can be contextualized within present-day theories of sensorimotor and cerebral processing.

As our results suggest an improvement to proprioception with the addition of a contra-lateral motor task. This could be partially explained through the theory that there is both hierarchical (cerebral) and parallel (peripheral) levels of processing of sensory information [53]. Moreover, it is thought that there is both a conscious (higher cerebral processing) and unconscious (peripheral reflexive processing) component to proprioceptive information [54]. Subsequently, it is entirely possible that proprioception information during the motor interference condition was appropriately interpreted at the peripheral level for monitoring and response execution [53]. Complementary to this line of thinking is the Multiple Resource Theory [55], which suggests that attentional resources are divided into different energy “pools”; for example, tasks that include a manual response use different resources than tasks that include a cognitive response [36].

Potentially our results are supporting the idea of a bimanual coupling effect, where there is a dependency between the upper limbs, which induces a mutual interference process [56]. This suggests that the motor performance of one limb could influence the motor performance of the other, sharing feedforward and feedback information. It is possible that during proprioceptive testing, the contralateral motor task was acting as a motor facilitator, effectively enhancing the sense of proprioception in the other limb or causing an increased neural drive of the nervous system. Along this same line of thinking, there could also be a similar excitability effect (or decreased inhibition) from the descending signals from the brain to the reflex arc, as seen with the Jendrassik Manoeuvre. The Jendrassik Manoeuvre is used in clinical practice during the testing of peripheral reflexes, to reinforce the reflexes by having an effect on the stretch, or H-reflex, and surrounding muscular activity. This is generally achieved by encouraging the patient to “hook their fingers together or clench their teeth” for example, reinforcing the monosynaptic reflexes along the descending corticospinal pathways, effectively exaggerating the reflex [57]. Although it is important to note that the underlying mechanism of this reinforcement has not been fully identified [57]. This type of excitability effect, or decreased inhibition along the corticospinal pathways, could explain why our results suggest a predominantly better proprioception performance during the simultaneous contralateral motor task.

On the other hand, the cognitive interference task caused a global worsening of proprioception when measured with the PRO-Reach, but an improvement to proprioception was observed during testing with the Biodex. This could suggest that participants made individual choices as to which task they prioritized [31]. During the PRO-Reach evaluation, participants could have prioritized the cognitive interference task, resulting in a worse sense of proprioception. Whereas during the Biodex evaluation, the proprioception testing could have been prioritized over the cognitive interference task. Interestingly, the proprioceptive testing with the Biodex saw an overall improvement to proprioception, during both interference conditions. This could be because the Biodex evaluation was more demanding of attentional resources for participants. It would have been interesting to ask participants which proprioceptive outcome they found more challenging, the Biodex or PRO-Reach.

### How is attention affected by dual-interference with an upper limb proprioception task?

When we decide to divide our attention among several activities (multi-tasking), we also need to choose how to engage our attention during motor performances [16]. During dual-task conditions, individuals can prioritize a specific component (interference or proprioception, for example), or it is also possible for deficits to be present in both tasks (mutual interference) [23]. From our findings, the overall number of attentional errors was quite low, suggesting that the participants performed the interference tasks well and allocated attentional resources to the interference tasks. Due to the mixed results seen with the magnitude of the proprioception errors during cognitive interference, this strengthens the theory that each participant choose their own attentional resource strategy [31]. It is entirely feasible that some prioritized the interference task, others the proprioception task while some attempted to divide their attention between the two tasks resulting in a mutual interference effect [23].

### Is there a relationship between upper limb proprioception and attention during dual-task interference?

As there were no strong correlational relationships between the proprioception errors and the number of attentional errors made, there does not appear to be a clear relationship between conscious upper limb proprioception and the allocation of attentional resources. A strong correlation between the number of PEs and the number of attentional errors could suggest that the entire CNS was simultaneously overloaded for both tasks. For example, still having plenty of attentional resources available, one participant could have made fewer PEs and fewer attentional errors, while another, having an attentional overload, would have made larger PEs and many attentional errors. Our results suggest that proprioception and the allocation of attention do not compete for the same resources, supporting the idea of sensorimotor processing occurring at both cerebral and peripheral levels, or within different “pools”. As most dual-task studies investigating proprioception involved the lower limbs and walking [51, 58, 59], we encourage further investigation with the upper limbs as the projections of the afferent pathways are different [60].

### Strengths and limitations

Our study is among the first to evaluate how interference influences conscious upper limb proprioception; more precisely, how cognitive and motor interference impacts AJPS. Although there appears to be a consensus that attention is not a unitary process [29], there is no agreement on the nomenclature used to describe attention or interference at this time [61]. Therefore, a strength of this study was the clear and meticulous definition of our interference outcomes, with a strong level of agreement between evaluators.

There are also limitations to be considered. As our study was performed with healthy young adults who were for the majority right-handed (90%), this topic requires further investigation as to the effect of dominance. It is understood that left-handed individuals can have different motor planning strategies and more pronounced bilaterality of cognitive processing [62], which could influence how they prioritize tasks. It is also worth reproducing this study with individuals experiencing upper limb pain, as the results may be different with this population. We encourage further research on the allocation of attention during upper limb AJPS testing, specifically among pathological and older populations [22]. We strongly recommend that further studies (e.g., a comparison of participants’ age, upper limb pathologies, and controlling for the effects of dominance) should be performed to better understand the impact of interference on upper limb proprioception. Also, it would have been interesting to concurrently evaluate other kinematic variables (such as reaction time, three dimensional trajectories of movement or limb speed) during proprioceptive testing. This would have allowed us to expose any compensatory strategies used by participants during dual-task testing.

Additionally, it is crucial to acknowledge that the PRO-Reach tool and Biodex System may assess different aspects of conscious upper limb proprioception. The Biodex evaluation was conducted while the participant was seated and focused on the glenohumeral joint with one degree of freedom, primarily assessing rotational movements. By contrast, the PRO-Reach tool assessed multi-joint movements of the entire upper limb, including trunk movements during reaching while standing. We cannot discount the possibility that the two upper limb proprioception outcomes examine different aspects and areas of proprioception acuity.

Lastly, it would have been beneficial to evaluate the participants’ ability to divide their attention, by using the Train-Making Test (parts A and B), which are used to examine the ability to alternate attention between two tasks [63].

## Conclusion

Our study revealed mixed results with cognitive interference, whereas the motor interference condition demonstrated a tendency for improving conscious upper limb proprioception. Our results support the theory of different levels of processing, for example hierarchical (CNS) and in parallel (PNS), while treating different sensory inputs and competing information within different attentional “pools”. There also appears to be an individual choice for task prioritization, where each person chooses their own attention strategy to navigate dual-task interference. We encourage further exploration of the effects of dual-task interference on upper limb AJPS by reproducing this study with pathological populations and the elderly. This line of inquiry has merit, as there are potential clinical applications for dual-task interference training for the rehabilitation of upper limb proprioception deficits.

## Supporting information

S1 File Correlational analysis proprioception and interference errors as well as laboratory protocols for the Biodex and PRO-Reach outcomes. (DOCX)
