# Supplementary material for: How does a motor or cognitive dual-task affect our sense of upper limb proprioception?
Source: PLoS One. 2024 Mar 20;19(3):e0299856. doi: 10.1371/journal.pone.0299856 (PMC10954121; doi:10.1371/journal.pone.0299856)
Supplement: S1 File — (DOCX) [file pone.0299856.s001.docx]

**Supplementary Table 1:** Correlational analysis between global mean proprioception errors and number of cognitive and motor errors (attentional errors)

| **Pearson’s Correlations Variables (Bivariate)** | **Findings and significance** |
| --- | --- |
| **Biodex Systems III** | |
| **Global mean PE** (two criterion positions) versus number of **cognitive errors** | *r* (40) = .284, *p* = .068 |
| **Global mean PE** (two criterion positions) versus number of **motor errors** | *r* (40) = .244, *p* = .120 |
| **PRO-Reach** | |
| **Global mean PE** (seven targets) versus number of **cognitive errors** | *r* (40) = .117, *p* = .460 |
| **Global mean PE** (seven targets) versus number of **motor errors** | *r (*40) = .095, *p* = .551 |

**Supplementary Table 2:** Mean count of attentional errors made during single and dual-task testing

| Proprioception Outcome | Cognitive Errors | Motor Errors |
| --- | --- | --- |
| Baseline – prior to proprioception testing (single task) | | |
|  | **# of numbers counted**  **in one minute**  38.9 ± 11.6  [14 – 73] | **# of hand movements**  **in one minute**  55.4 ± 24.8  [23 - 128] |
|  | **# errors made**  6.4 ± 5.6  [0 – 29] | **# errors made**  0.7 ± 2.1  [0 – 10] |
|  |  |  |
| During proprioception testing (dual-task) | | |
| Biodex Systems (AJPS errors in °) | | |
| AJPS 75% | 0.9 ± 0.9  [0 – 4.0) | 1.3 ± 1.3  [0 – 4.7] |
| AJPS 90% | 0.7 ± 0.7  [0 – 3.2] | 1.3 ± 1.5  [0 – 5.0] |
| PRO-Reach (AJPS errors in cm) | | |
| S | 0.3 ± 0.4  [0 – 1.2] | 0.8 ± 0.8  [0 – 3.0] |
| SLD | 0.3 ± 0.3  [0 – 0.8] | 0.8 ± 0.6  [0 – 2.7] |
| LD | 0.3 ± 0.4  [0 – 1.3] | 0.7 ± 0.8  [0 – 3.8] |
| ILD | 0.4 ± 0.4  [0 – 1.7] | 0.7 ± 0.6  [0 – 3.0] |
| SLND | 0.3 ± 0.4  [0 – 1.2] | 0.8 ± 0.8  [0 – 3.0] |
| LND | 0.4 ± 4.5  [0 – 1.7] | 0.9 ± 0.9  [0 – 4.5] |
| ILND | 0.3 ± 0.4  [0 – 1.2] | 0.8 ± 0.9  [0 – 3.8] |
| Global mean  (7 targets) | 0.3 ± 0.3  [0 – 1.0] | 0.8 ± 0.7  [0 – 3.2] |

Baseline attentional errors assessed before the evaluation of proprioception (no proprioception interference) and during proprioception testing (dual-task interference).

The cognitive task comprised of counting backwards from 100 by threes. An error was counted if the participant did not state the correct number, if there was a word whisker (errr, ummm), or if there was a pause longer than 2 seconds.

The motor task included opening and closing of their non-dominant hand. An error was counted if the participant was unable to fully extend or flex their digits or thumb, or if their movements stopped for longer than 2 seconds.

The mean number of attentional errors, as assessed by two evaluators, during the simultaneous proprioception testing of the Biodex or PRO-Reach (dual-task interference).

AJPS = active joint position sense, PRO-Reach = Upper Limb Proprioception Reaching Test

Biodex Systems = Isokinetic Dynamometer, Biodex Systems III.

Descriptive statistics of attentional errors, Mean $\pm$ SD [min – max], of healthy participants (n=42)

PRO-Reach targets: Superior (S), Superior Lateral Dominant (SLD), Lateral Dominant (LD),

Inferior Lateral Dominant (ILD), Superior Lateral Non-Dominant (SLND), Lateral Non-Dominant (LND) and Inferior Lateral Non-Dominant (ILND).

Participant : ________________________________

Evaluation Date ( day/month/year ) : / / .

**UPPER LIMB PROPRIOCEPTION REACHING TEST (PRO-Reach)**

Dominant shoulder: L / R

Equipment:

- PRO-Reach tool
- Adhesive magnet strips
- Tape measure
- Numbered stickers (1-3)
- Measuring Tape (visible mm)
- Alcohol wipes / cleaner
- Clipboard + pen

Participant distance from the wall (Wall to 1st toe): ________________ cm

Pain at rest BEFORE evaluation: __________ / 10

*From 0 to 10, 0 being no pain, 10 being the worst pain of your life, how is your shoulder feeling at this time?*

**DOMINANT LIMB**

| **Order**  **Randomized** | **Direction** | **Trial 1**  **(cm)** | **Trial 2**  **(cm)** | **Trial 3**  **(cm)** |
| --- | --- | --- | --- | --- |
|  | **Superior** |  |  |  |
|  | **SLD** |  |  |  |
|  | **LD** |  |  |  |
|  | **ILD** |  |  |  |

**NON-DOMINANT LIMB**

| **Order**  **Randomized** | **Direction** | **Trial 1**  **(cm)** | **Trial 2**  **(cm)** | **Trial 3**  **(cm)** |
| --- | --- | --- | --- | --- |
|  | **Superior** |  |  |  |
|  | **SLND** |  |  |  |
|  | **LND** |  |  |  |
|  | **ILND** |  |  |  |

Pain at rest AFTER evaluation: __________ / 10

*From 0 to 10, 0 being no pain, 10 being the worst pain of your life, how is your dominant shoulder feeling at this time?*

**Evaluator**: _____________________________________


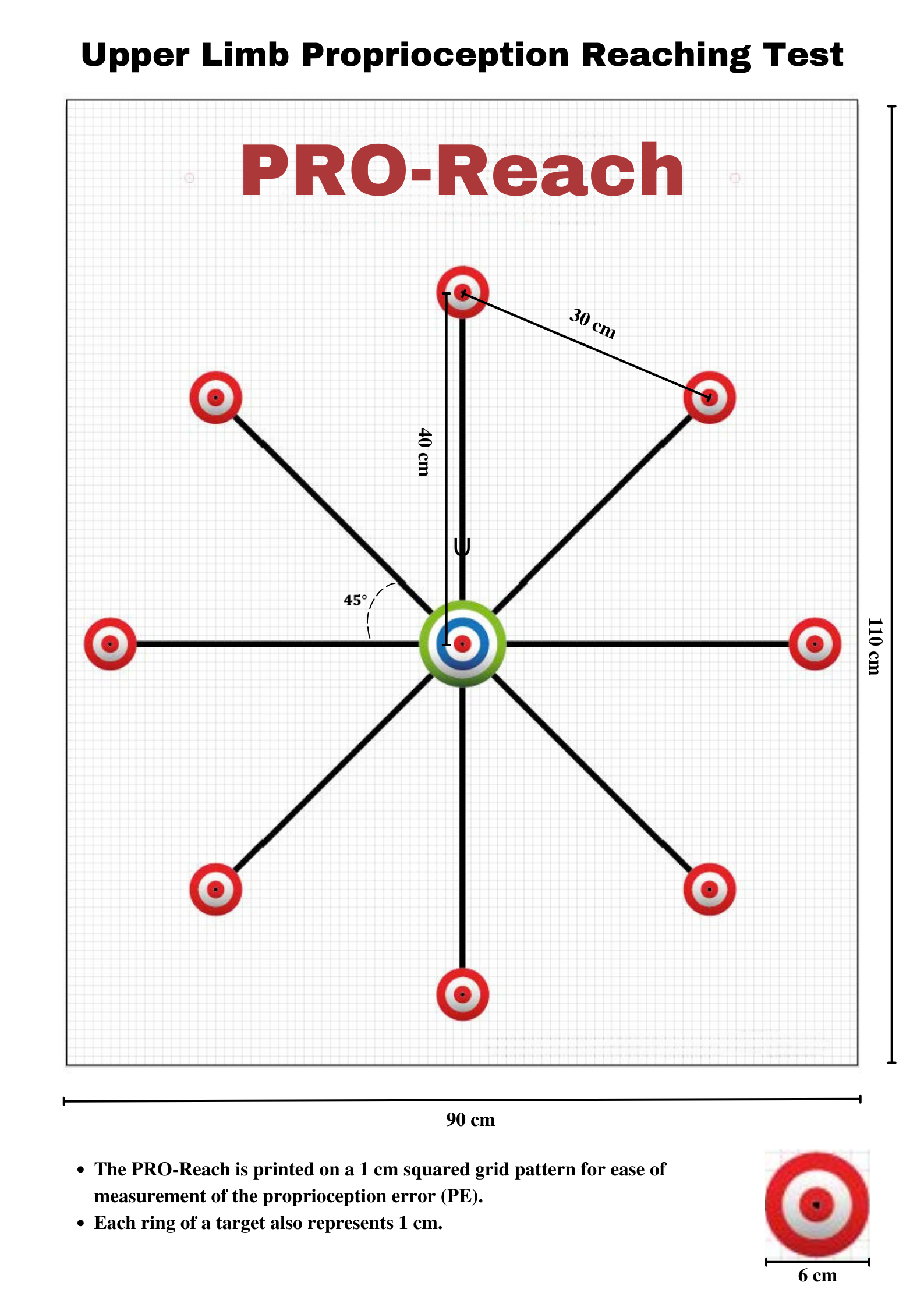


**Measured shoulder range of motion (ROM) per target**

| Target | Measured shoulder movements  from pilot testing (n=10) |
| --- | --- |
| Superior  (S) | 130$^{\circ}\pm10^{\circ}$ forward flexion |
| Superior Lateral Dominant  (SLD) | 120$^{\circ}\pm10^{\circ}$ flexion with 25$^{\circ}\pm10^{\circ}$ lateral deviation |
| Lateral Dominant  (LD) | 90$^{\circ}\pm10^{\circ}$ flexion with 40$^{\circ}\pm10^{\circ}$ lateral deviation |
| Inferior Lateral Dominant  (ILD) | 70$^{\circ}\pm10^{\circ}$ flexion with 25$^{\circ}\pm10^{\circ}$ lateral deviation |
| Superior Lateral Non-Dominant (SLND) | 120$^{\circ}\pm10^{\circ}$ flexion with 25$^{\circ}\pm10^{\circ}$ medial deviation (cross-body movement) |
| Lateral Non-Dominant  (LND) | 90$^{\circ}\pm10^{\circ}$ flexion with 25$^{\circ}\pm10^{\circ}$ medial deviation (cross-body movement) |
| Inferior Lateral Non-Dominant (ILND) | 70$^{\circ}$ flexion with 25$^{\circ}\pm10^{\circ}$ medial deviation  (cross-body movement) |

***Caption:*** *The Upper Limb Proprioception Reaching Tool (PRO-Reach) measures the resultant proprioception error (PE) in centimeters, between the centre of the target, and the resultant reproduced movement by the participant (where their dominant index finger makes contact with the PRO-Reach). The trajectory of the movement is not measured. The measured shoulder range of motion represented in this table reflects pilot testing from ten healthy Master students. The presented range of motion reflects the final reproduced shoulder movement, with a 10*$^{\circ}$ *latitude, representing the variability between evaluated participants.*

**The Upper Limb Proprioception Reaching Test (PRO-Reach)**

**Purpose:** To measure the active joint position sense (AJPS) (proprioception error [PE] in centimetres) of the upper limb through a functional and active reaching movement in standing.

**Instructions for the Evaluator:**

The evaluator positions the participant facing the PRO-Reach with their arm at 90° of shoulder flexion, verified using a goniometer. The height of the PRO-Reach is adjusted so that their dominant arm is at the height of the central target (multi-coloured target in the centre), with their 3^rd^ digit touching the center of the target. Once adjusted, the distance from the wall to the hallux is measured to standardize the position for the participant, and marked with a piece of tape. The participants are not to move their feet during the evaluation (their feet hip-width apart, in an overall neutral posture).

The order of evaluation for the targets are randomized.

The participant is instructed to point their index finger and place it in the centre of the target with eyes open. Once the index finger makes contact with the centre of the target, the participant closes their eyes and memorizes their position for five seconds. The evaluator counts softly with a monotone voice while observing a timer, at which point the participant returns their arm to their side and opened their eyes. This is repeated three times, as the “memorization trials”.

Immediately following the memorization trials, the participant lowers their blindfold resting on their forehead with the non-evaluated arm and reproduced the memorized position three times. No feedback or corrections are given during testing. After each trial, the evaluator places a numbered sticker (1 through 3) immediately above the nail of the index finger, without making contact.

When the participant starts the reaching trials, you are no longer speaking to them, you must be quiet throughout the memorization and reproduction trials. Also, you place the sticker at the first location where the index finger touches the mat. The sticker is placed immediately above their nail (as close as possible, without making contact with the participant). This process is repeated for all seven targets. Each target will have three PEs to measure, at the end of the evaluation (from the centre of the sticker, to the centre of the target being evaluated).

**Evaluation Protocol:**

- The participant will face the PRO-Reach tool mounted on the wall with adhesive magnets. The participant is standing with their feet hip-width apart, in an overall neutral posture.
- The evaluator will adjust the mat so that the center of the glenohumeral (GH) joint of the participant is centered on the PRO-Reach tool, indicated by the red central circle. This is done by asking the participant to reach for the central red target (mid-supination / pronation of the arm), so that the tip of their middle finger touches the target comfortably. The distance from the 1^st^ toe to the wall will be measured and noted by the evaluator on the evaluation sheet.
- Participants will then actively practice with their eyes open, three reaching trials towards the seven targets, in a randomized ordered determined by a non-partial evaluator.
- The ordered will be determined by pulling the various directions from an opaque envelop.
- The participant will attempt to reproduce the exact movement practiced with his/her eyes open. The attempts towards the targets will be measured with their eyes closed. This represents the proprioception error (PE) in centimetres.
- The participants will be asked to “MEMORIZE THE POSITION OF YOUR ARM IN SPACE". They will have three sequential practices, where they have their eyes open during the movement towards the target, and instructed to close their eyes, once their index finger makes contact with the PRO-Reach tool.
- The evaluator will then count in a low and monotone voice, 1 through 5, and say "Okay" when the participant is to return their arm to the side of their body.
- Following 3 memorization trials, the participant is to lower their blindfold (sitting on their forehead) with the non-evaluator arm, and immediately reproduced the practiced movement three times in a row. No feedback, disruptions or breaks are to occur during the three replication trials.
- The evaluator will mark the tip of the index finger after each trial with a numbered sticker (1 through 3).
- The sticker is to be placed as close to the nail of the index finger, without touching the participant.
- The process will be repeated for each target, Superior (S) / Superior Lateral Dominant (SLD) / Superior Lateral Non-Dominant (SLND) / Lateral Dominant (LD) / Lateral Non-Dominant (LND) / Inferior Lateral Dominant (ILD) and Inferior Lateral Non-Dominant (ILND). The difference (in centimetres, to one decimal point) between the target and the tip of the index finger (the centre of the applied sticker) of each trial represents the Proprioceptive Error (PE).

**Instructions for the Participant:**

**Dress:**

Barefoot (to measure the distance from the hallux to the wall)

**Males:** bare-chested, hair off the shoulders, no jewellery or watches on the upper limbs

**Females:** sports bra or thin strapped camisole, no jewellery or watches on the upper limbs

The evaluator will first provide all directions clearly and answer questions from the participant. The evaluator will demonstrate the entire protocol on the superior target. The participant will then have the opportunity to practice the entire protocol (3 memorization and 3 reproduction trials), also on the superior target. A break of 5 minutes is given between the explanation and the commencement of the evaluation. No talking or questions are permitted during testing.

1. Stand facing the mat. The evaluator will centre your shoulder on the red circle.
2. Using your dominant arm, reach towards the target in the superior direction.
3. When your index finger makes contact with the tool, close your eyes, and MEMORIZE THE POSITION OF YOUR ARM IN SPACE. (Evaluator counts to 5, slowly and calmly) OKAY. (Participant returns arm along the side of the body).
4. You will have three practice trials, and then three reproduction trials.
5. Immediately following this practice, YOU WILL LOWER YOUR BLINDFOLD WITH YOUR OPPOSITE HAND, AND REPRODUCE THE EXACT MOVEMENT YOU JUST PRACTICED. You will do this three times in a row.
6. You will be given a 5 second break before you change targets.
7. Lastly - once your index finger touches the mat, you must stay in the position. Your finger is not permitted to move once it has made contact with the mat.
8. Do you have any questions?
9. On a scale from 0 to 10, 0 representing no pain at all, and 10 the worst pain of your life, are you currently experiencing any pain to your shoulder?

If an error is made during the testing of a specific target, the target is to be re-evaluated (all three trials) at the end of the entire evaluation, following a 5-minute rest period.

**An error includes:**

- Significant noise or distraction during the evaluation;
- A displacement of the participant’s feet during the evaluation;
- The blindfold does not remain in place and vision is restored;
- The index finger does not stay on the location where it made initial contact with the PRO-Reach poster (for example, the finger touches the poster, but continues moving along the plasticised poster);
- The evaluator makes physical contact with the participant, when placing the sticker above their index finger.

The PRO-Reach tool is to be cleaned with alcohol wipes between participants, to remove any residue from the evaluation stickers on the tool.

**Biodex Systems – Active Joint Position Sense**

**of the Dominant Shoulder**

Outcome: Active joint position sense (AJPS) is a subcategory of proprioception, and is among the most measured aspect of shoulder proprioception. As there are at present no Gold Standard for the measurement of AJPS of the shoulder, the measurement of internal rotation with the Biodex Systems will be considered our Gold Standard for this study.

Equipment: Biodex Multijoint System IV (or Systems III); Biodex Medical Systems, Inc., Shirley, NY, USA

Position: Participant is tested in seated upright position and will be secured with pelvic and torso straps. The glenohumeral (GH) joint will be positions at 90° of abduction, and the elbow flexed to 90°. The forearm will be secured to the resistance arm, limiting motion to only internal or external rotation.

**Dress:**

**Males:** bare-chested, hair off the shoulders, no jewellery or watches on the upper limbs

**Females:** sports bra or thin strapped camisole, no jewellery or watches on the upper limbs

The evaluator will first provide all directions clearly and answer questions from the participant. The evaluator will demonstrate the entire protocol with the Biodex Arm. The participant will then have the opportunity to practice the entire protocol (3 memorization and 3 reproduction trials) at 50% of their maximum internal rotation. A break of 5 minutes is given between the explanation and the commencement of the evaluation. No talking or questions are permitted during testing.

Protocol:

- Reference starting position: 90° of shoulder abduction, 0° of IR / ER (neutral).
- Internal rotation (IR) at 90° of shoulder abduction will be tested, with two relative target angles (criterion positions, 75% and 90% of IR) to reproduce. The angles will be actively performed and the participant will be told to stop by the Biodex at the correct relative angle.
- The directions and relative angles will be randomized.
- After the practice round, the participants will actively reproduce the reference angles and told to hold reach reference angle for 5 seconds. The participants will be told to “concentrate on the position of the arm in space”.
- This will be done three times for each criterion position. The order of the criterion positions is randomized.
- The participant will be given 5 seconds to concentrate on the presented reference position. The arm will then be passively rotated back by the Biodex to the starting position, at a speed of 0.5°/s.

Outcome recorded: The angles (6 measurements total) reproduced by the participant, to subsequently calculate the absolute, exact, and variable error for statistical analysis.

**Directives for testing AJPS of the participants:**

- Now, we are going to be testing your sense of where your arm is in space. We will be performing three sets of trial in this direction.
- We will first bring you to specific movement, where you will be asked to **HOLD** the position, with a gentle contraction of your muscles. You will memorize this position for 5 seconds.
- Then, you will actively bring your arm to that same target position.
- When you feel you have reached the target position, you will push the button in your other hand.
- We will repeat this process 3 times for this movement. Always a practice, and then you reproducing the angle.
- You will hear the following commands from me: **MOVE TO TARGET, HOLD, RELAX**.
- You will be asked to hold and memorize a position. We will bring your arm back to the starting position.
- You will actively reproduce the movement yourself, when I say: **READY, GO**.
- Bring yourself to the target angle, and again press the black button in your other hand.
- You will hear the following commands from me: **READY, GO. HOLD, RELAX**.

Identification number : ________________________________

Evaluation Date ( day/month/year ) : / / .

**AJPS with the Biodex Systems**

Evaluation of dominant shoulder: R / L

Pain at rest BEFORE evaluation: __________ / 10

*From 0 to 10, 0 being no pain, 10 being the worst pain of your life, how is your dominant shoulder feeling at this time?*

**ACTIVE JOINT POSITION SENSE (DOMINANT SHOULDER)**

Max end-range in **ACTIVE** Internal **Rotation (IR)** at 90° of abduction: ______________________

**Active Joint Position Sense (AJPS)**

| **Testing Angles** | **Trial 1** | | **Trial 2** | | **Trial 3** | | |  |
| --- | --- | --- | --- | --- | --- | --- | --- | --- |
|  | **True**  **Angle** | **Relative Angle** | **True**  **Angle** | **Relative Angle** | | **True**  **Angle** | **Relative Angle** | |
| **75% IR** |  |  |  |  | |  |  | |
| **90% IR** |  |  |  |  | |  |  | |

*N.B. Indicate (+) for an over estimation, (-) for an under estimation.*

Pain at rest AFTER evaluation: __________ / 10

*From 0 to 10, 0 being no pain, 10 being the worst pain of your life, how is your dominant shoulder feeling at this time?*

**Evaluator**: _____________________________________ **Date**: _______________________
